# Obese patients who fall have less injury severity but a longer hospital stay than normal-weight patients

**DOI:** 10.1186/s13017-015-0059-9

**Published:** 2016-01-04

**Authors:** Jung-Fang Chuang, Cheng-Shyuan Rau, Hang-Tsung Liu, Shao-Chun Wu, Yi-Chun Chen, Shiun-Yuan Hsu, Hsiao-Yun Hsieh, Ching-Hua Hsieh

**Affiliations:** Department of Trauma Surgery, Kaohsiung Chang Gung Memorial Hospital and Chang Gung University College of Medicine, No.123, Ta-Pei Road, Niao-Sung District, Kaohsiung City, 833 Taiwan; Department of Neurosurgery, Kaohsiung Chang Gung Memorial Hospital and Chang Gung University College of Medicine, Kaohsiung, Taiwan; Department of Anesthesiology, Kaohsiung Chang Gung Memorial Hospital and Chang Gung University College of Medicine, Kaohsiung, Taiwan

**Keywords:** Fall, Obesity, Injury severity score, Mortality, In-hospital length of stay

## Abstract

**Background:**

The effects of obesity on injury severity and outcome have been studied in trauma patients but not in those who have experienced a fall. The aim of this study was to compare injury patterns, injury severities, mortality rates, and in-hospital or intensive care unit (ICU) length of stay (LOS) between obese and normal-weight patients following a fall.

**Methods:**

Detailed data were retrieved for 273 fall-related hospitalized obese adult patients with a body mass index (BMI) ≥30 kg/m^2^ and 2357 normal-weight patients with a BMI <25 kg/m^2^ but ≥18.5 kg/m^2^ from the Trauma Registry System of a Level I trauma center between January 1, 2009, and December 31, 2013. We used the Pearson’s chi-squared test, Fisher’s exact test, the Mann Whitney *U* test, and independent Student’s *t*-test to analyze differences between the two groups.

**Results:**

Analysis of AIS scores and AIS severity scaling from 1 to 5 revealed no significant differences in trauma regions between obese and normal-weight patients. When stratified by injury severity (Injury Severity Score [ISS] of <16, 16–24, or ≥25), more obese patients had an ISS of <16 compared to normal-weight patients (90.5 % vs. 86.0 %, respectively; *p* = 0.041), while more normal-weight patients had an ISS between 16 and 24 (11.0 % vs. 6.6 %, respectively; *p* = 0.025). Obese patients who had experienced a fall had a significantly lower ISS (median (range): 9 (1–45) *vs.* 9 (1–50), respectively; *p* = 0.015) but longer in-hospital LOS than did normal-weight patients (10.1 days *vs.* 8.9 days, respectively; *p* = 0.049). Even after taking account of possible differences in comorbidity and ISS, the obese patients have an average 1.54 day longer LOS than that of normal-weight patients. However, no significant differences were found between obese and normal-weight patients in terms of the New Injury Severity Score (NISS), Trauma-Injury Severity Score (TRISS), mortality, percentage of patients admitted to the ICU, or LOS in the ICU.

**Conclusion:**

Obese patients who had experienced a fall did not have different injured body regions than did normal-weight patients. However, they had a lower ISS but a longer in-hospital LOS than did normal-weight patients.

## Background

Falls are a leading cause of injury and a significant public health issue [1–3]. The incidence of falls that lead to emergency unit admission is growing with the increased size and rapid growth of the geriatric population [4, 5]. In addition, obesity is a worldwide health problem leading to a range of health consequences [6, 7]. While obesity is known to increase the risk for a variety of medical conditions including hypertension, diabetes mellitus, cardiac disease, and pulmonary thromboembolism [8], the effect of obesity on the injury pattern and outcome of trauma patients after a fall remains unclear. Evidence was found that the effect of weight on the risk of falling appeared to be linear; greater obesity was related to greater risk of falling [9–11]. Compared with normal-weight respondents, the odds ratios (OR) for risk of falling were 1.12 (95 % confidence interval [CI] = 1.01–1.24) for obesity Class 1 (BMI 30.0–34.9 kg/m^2^), 1.26 (95 % CI = 1.05–1.51) for obesity Class 2 (BMI 35.0–39.9 kg/m^2^), and 1.50 (95 % CI = 1.21–1.86) for obesity Class 3 (BMI ≥ 40.0 kg/m^2^) [9]. In addition, obesity was associated with a 25 % higher risk (95 % CI = 1.11–1.41; *p* < 0.0003) of having fallen in the previous 12 months compared to non-obese individuals [12].

Identification of the high-risk injury patterns and better understanding of the epidemiology and outcome of fall injury in obese patients are important in order to cope with a rising number of obese patients. Therefore, this study was designed to investigate the injury characteristics, injury patterns, injury severities, and mortality rates of adult obese patients admitted and treated for fall-related injury in southern Taiwan over a five-year period at a level I trauma center.

## Methods

### Ethics statement

Approval for this study was obtained from the hospital’s institutional review board (IRB) before its initiation (approval number 103-7110B). Given its observational nature, the requirement for written informed consent from each patient was waived by the IRB.

### Study design

This retrospective study was designed to review all 16,548 hospitalized and registered patients added to the Trauma Registry System from January 1, 2009, to December 31, 2013, and select cases that met the following inclusion criteria: (1) age ≥ 18 years, (2) BMI ≥ 30 kg/m^2^ for obese patients and BMI < 25 but ≥ 18.5 kg/m^2^ for normal-weight patients according to the World Health Organization definition [13, 14], and (3) admittance due to a fall accident. The patients who had sustained fall injuries from all fall heights (<1 m, 1–6 m, >6 m) were included, but those who had attempted suicide in the fall or who had non-validated BMI values or incomplete data were excluded.

To compare the injury patterns, mechanisms, severity, and mortality of obese patients with those of normal-weight patients, detailed data were retrieved on age, sex, vital signs in the emergency department (ED), injury mechanism, fall height (<1 m, 1–6 m, >6 m), transportation, injury time, Glasgow Coma Scale (GCS) upon arrival at the ED, Abbreviated Injury Scale (AIS) severity score for each body region, Injury Severity Score (ISS), New Injury Severity Score (NISS), Trauma-Injury Severity Score (TRISS), in-hospital length of stay (LOS), LOS in the ICU, and in-hospital mortality. In addition, the pre-existed comorbidities and chronic diesases including diabetes mellitus (DM), hypertension (HTN), coronary artery diseases (CAD), congestive heart failure (CHF), cerebrovascular accident (CVA), and end-stage renal disease (ESRD) were identified. A blood alcohol concentration (BAC) of 50 mg/dL at the time of arrival at the hospital was defined as the cut-off value according to the legal limit for drivers in Taiwan. The primary outcomes were injury severity scores (i.e., GCS, AIS, ISS, NISS, and TRISS), and the secondary outcomes were LOS, ICU LOS, and in-hospital mortality.

The ORs of the injured regions and associated conditions sustained by obese and normal-weight patients were calculated with 95 % CIs. Data collected regarding the obese and normal-weight population of patients who had experienced a fall were compared using SPSS v.20 statistical software (IBM, Armonk, NY, USA). Pearson’s chi-squared tests, Fisher’s exact tests, and independent Student’s t-tests were used to analyze data as applicable. The Mann Whitney *U* test was used to compare the AIS severity scaling from 1 to 5 in each injury region. Ordinal data, like ISS and NISS, is presented as median (range). Data were further analyzed by a multiple linear regression adjusted for the effect of confounding variables (ie, comorbidity and ISS) to show the main effects of obesity on LOS in hospital. All other results are presented as the mean ± standard error.

## Results

### Injury characteristics

Among the 2630 adult patients with fall accidents, 273 (10.4 %) were obese (BMI ≥ 30 kg/m^2^), and 2357 (89.6 %) were of normal weight (25 > BMI ≥ 18.5 kg/m^2^) (Table 1). No statistically significant difference regarding sex was found between the obese and normal-weight patients. The mean ages of the obese and normal-weight patients were 60.6 ± 16.8 and 65.7 ± 17.1 years, respectively (*p* <0.001). There were significant higher incidence rates of the pre-existed comorbidities and chronic diseases including DM, HTN, and CAD in the obese patients. The majority of patients in both groups fell from a height < 1 m, implying that the majority of the patients sustained a ground-level fall occurring upon walking or with movement; however, this difference in patient number stratified by fall height (<1 m, 1–6 m, >6 m) was not statistically significant.

**Table 1:** Demographics of the obese and normal-weight patients with a fall injury

Variables	Obese	Normal	*Odds ratio (95 %)*	*P*
	BMI ≥ 30	25 > BMI ≥ 18.5		
	*n* = 273	*n* = 2357		
Gender				
Male	114(41.8)	1080(45.8)	0.8(0.66–1.09)	0.202
Female	159(58.2)	1277(54.2)	1.2(0.92–1.52)	0.202
Age	60.6 ± 16.8	65.7 ± 17.1	—	<0.001
Comorbidity				
DM	98(35.9)	497(21.1)	2.1(1.61–2.74)	<0.001
HTN	153(56.0)	995(42.2)	1.7(1.36–2.25)	<0.001
CAD	26(9.5)	137(5.8)	1.7(1.10–2.65)	0.016
CHF	8(2.9)	51(2.2)	1.4(0.64–2.91)	0.418
CVA	25(9.2)	216(9.2)	1.0(0.65–1.54)	0.997
ESRD	0(0.0)	7(0.3)	—	1.000
Height of fall				
< 1 m	213(78.0)	1859(78.9)	1.0(0.70–1.29)	0.745
1–6 m	59(21.6)	471(20.0)	1.1(0.81–1.50)	0.525
> 6 m	1(0.4)	27(1.1)	0.3(0.04–2.34)	0.354
Alcohol > 50, n(%)	4(1.5)	51(2.2)	0.7(0.24–1.88)	0.445
GCS	14.6 ± 1.7	14.4 ± 2.0	—	0.104
≤ 8	8(2.9)	80(3.4)	0.9(0.41–1.80)	0.687
9–12	6(2.2)	88(3.7)	0.6(0.25–1.34)	0.196
≥ 13	259(94.9)	2189(92.9)	1.4(0.81–2.49)	0.218
AIS, n(%)				
Head/Neck	48(17.6)	537(22.8)	0.7(0.52–1.00)	0.050
Face	12(4.4)	154(6.5)	0.7(0.36–1.20)	0.169
Thorax	20(7.3)	174(7.4)	1.0(0.61–1.60)	0.973
Abdomen	16(5.9)	131(5.6)	1.1(0.62–1.81)	0.837
Extremity	217(79.5)	1792(76.0)	1.2(0.90–1.66)	0.203
ISS, median(range)	9(1–45)	9(1–50)	—	0.015
< 16	247(90.5)	2027(86.0)	1.5(1.02–2.36)	0.041
16–24	18(6.6)	259(11.0)	0.6(0.35–0.94)	0.025
≥ 25	8(2.9)	71(3.0)	1.0(0.46–2.04)	0.940
NISS, median(range)	9(1–66)	9(1–75)	—	0.070
TRISS	0.960 ± 0.112	0.958 ± 0.085	—	0.645
Mortality, n(%)	7(2.6)	55(2.3)	1.1(0.50–2.44)	0.812
Height of fall				
< 1 m	4(1.5)	44(1.9)	0.8(0.28–2.19)	0.639
1–6 m	3(1.1)	11(0.5)	2.4(0.66–8.55)	0.171
LOS (days)	10.1 ± 10.3	8.9 ± 8.9	—	0.049
Controlled by Comorbidity & ISS	—	—	1.01(1.01–1.03)	0.004
ICU				
Patients, n(%)	33(12.1)	375(15.9)	0.7(0.50–1.06)	0.099
< 16	15(5.5)	145(6.2)	0.9(0.51–1.53)	0.667
16–24	11(4.0)	167(7.1)	0.6(0.30–1.03)	0.057
≥ 25	7(2.6)	63(2.7)	1.0(0.43–2.11)	0.916
LOS in ICU (days)	8.2 ± 8.8	7.8 ± 9.6	—	0.833
< 16	6.6 ± 6.7	6.3 ± 8.7	—	0.908
16-24	7.5 ± 6.6	7.7 ± 10.0	—	0.963
≥ 25	11.9 ± 12.5	13.3 ± 13.4	—	0.782
AIS ≥3 sites, n(%)	6(0.2)	78(3.3)	0.7(0.28–1.52)	0.323
Mortality	0(0.0)	0(0.0)	—	—
LOS (days)	16.2 ± 10.2	14.8 ± 12.2	—	0.784
ICU Patients, n(%)	3(50.0)	36(46.2)	1.2(0.22–6.14)	1.000
LOS in ICU (days)	11.3 ± 9.1	7.1 ± 6.4	—	0.293

### Injury severity

No significant differences were found between obese and normal-weight patients in GCS scores (14.6 ± 1.7 *vs.* 14.4 ± 2.0, respectively; *p* = 0.104) and the distribution of the proportion of patients at different levels of consciousness (GCS ≤8, 9–12, or ≥13) (Table 1). Analysis of AIS scores revealed no significant differences in trauma regions between obese and normal-weight patients. Comparison of the composition of AIS severity scaling from 1 to 5 in each region between obese and normal-weight patients also did not show a significant difference (Table 2). A significant difference in ISS was found between obese and normal-weight patients (median (range): 9 (1–45) *vs.* 9 (1–50), respectively; *p* = 0.015). When stratified by injury severity (ISS of <16, 16–24, or ≥25), more obese than normal-weight patients had an ISS < 16 (90.5 % vs. 86.0 %, respectively; *p* = 0.041), while more normal-weight than obese patients had an ISS between 16 and 24 (11.0 % vs. 6.6 %, respectively; *p* = 0.025). However, no significant difference were found for NISS (median (range): 9 (1–66) *vs.* 9 (1–75), respectively; *p* = 0.070), TRISS (0.960 ± 0.112 *vs.* 0.958 ± 0.085, respectively; *p* = 0.645), or in-hospital mortality (2.6 % *vs*. 2.3 %, respectively; *p* = 0.645). We found that obese patients had a significantly longer average in-hospital LOS than did normal-weight patients (10.1 *vs.* 8.9 days, respectively; *p* = 0.049). Because the detected significant higher incidence rates of DM, HTN, and CAD in the obese patients or a higher ISS may be positively correlated to a longer hospital stay, therefore, we performed a multiple linear regression analysis to investigate the effect of obesity, DM, HTN, CAD, and ISS on LOS (days) of these patients. The analysis of variance table (Table 3) indicates that the relationship between obesity and LOS is significant (*p* = 0.005), LOS in obesity tends to be 1.54 day longer, on average, than that of normal-weight patients, even after taking account of possible differences in comorbidity and ISS. In addition, the relationship between ISS and LOS is highly significant (*p* < 0.0005), with a one score increase in ISS being associated with an average increase of 0.64 day LOS, after adjusting for obesity and comorbidity. However, no differences were noted in the proportion of obese and normal-weight patients admitted to the ICU as well as LOS in the ICU after stratification into either group of injury severity (ISS of <16, 16–24, or ≥25). In addition, among those who had sustained 3 or more body area injury (AIS ≥3 sites), there were no difference in obese and normal-weight patients admitted to the ICU, LOS in the ICU and in the hospital, and the mortality.

**Table 2:** Explanatory variables by a multiple regression analysis to investigate the effect of obesity, comorbidity, and ISS on LOS of these patients

Variables	Obese	Normal	Mann–Whitney *U* Test *P*
	BMI ≥ 30	25 > BMI ≥ 18.5	
	n(%)	n(%)	
Head/Neck	48	537	0.210
AIS 1	2(29.2)	112(20.9)	
AIS 2	4(8.3)	44(8.2)	
AIS 3	10(20.8)	104(19.4)	
AIS 4	15(31.3)	225(41.9)	
AIS 5	5(10.4)	52(9.7)	
Face	12	154	0.550
AIS 1	4(33.3)	71(46.1)	
AIS 2	8(66.7)	83(53.9)	
Thorax	20	176	0.804
AIS 1	4(20.0)	19(10.8)	
AIS 2	5(25.0)	56(31.8)	
AIS 3	5(25.0)	77(43.8)	
AIS 4	6(30.0)	22(12.5)	
AIS 5	0(0.0)	2(1.1)	
Abdomen	17	131	0.413
AIS 1	4(23.5)	6(4.6)	
AIS 2	7(41.2)	83(63.4)	
AIS 3	6(35.3)	40(30.5)	
AIS 4	0(0.0)	2(1.5)	
Extremity	217	1792	0.073
AIS 1	9(4.2)	43(2.4)	
AIS 2	86(39.6)	633(35.3)	
AIS 3	121(55.8)	1114(62.2)	
AIS 4	0(0.0)	2(0.1)	
AIS 5	1(0.5)	0(0.0)

**Table 3:** Comparison of the composition of AIS severity scaling from 1 to 5 in each region between obese and normal-weight patients

Variable	Parameter estimate	Standard error	95 % CI for parameter	*P*-value
Intercept	2.600	0.362	(1.891–3.310)	0.000
Obesity	1.543	0.544	(0.477–2.610)	0.005
DM	0.990	0.418	(0.170–1.809)	0.018
HTN	0.485	0.354	(−0.209–1.178)	0.171
CAD	0.962	0.690	(−0.392–2.315)	0.164
ISS	0.642	0.032	(0.579–0.705)	0.000

The multiple linear regression equation is given by: LOS (days) =2.60 + 1.54*(obesity) + 0.99*(DM) + 0.49*(HTN) + 0.96*(CAD) + ISS*0.64, depending on obesity (o = normal weight, 1 = obesity), DM (0 = no DM, 1 = DM), HTN (0 = no HTN, 1 = HTN), CAD (0 = no CAD, 1 = CAD), and ISS (scores) of these patients

### Physiological response & procedures performed at the ED

Upon arrival at the ED, no significant differences were found for GCS of <13, systolic blood pressure (SBP) of <90 mmHg, heart rate of >100 beats/min, or respiratory rates of <10 or >29. Furthermore, no significant differences were found in the odds for requiring procedures, including cardiopulmonary resuscitation, intubation, chest tube insertion, and blood transfusion, at the ED (Table 4).

**Table 4:** Physiological response and procedures performed upon arrival at the ED

Variables	Obese	Normal	*Odds ratio (95 %)*	*P*
	BMI ≥ 30	25 > BMI ≥ 18.5		
	*n* = 273	*n* = 2357		
Physiology at ER, n(%)				
GCS < 13	14(5.1)	168(7.1)	0.7(0.40–1.23)	0.218
SBP < 90 mmHg	3(1.1)	26(1.1)	1.0(0.30–3.31)	1.000
Heart rate > 100 beats/min	52(19.0)	357(15.1)	1.3(0.96–1.82)	0.092
Respiratory rate < 10 or >29	0(0.0)	6(0.3)	—	1.000
Procedures at ER, n(%)				
Cardiopulmonary resuscitation	0(0.0)	1(0.0)	—	1.000
Intubation	3(1.1)	34(1.4)	0.8(0.23–2.49)	1.000
Chest tube insertion	2(0.7)	23(1.0)	0.7(0.18–3.19)	1.000
Blood transfusion	8(2.9)	56(2.4)	1.2(0.59–2.63)	0.574

## Discussion

In a retrospective review of all blunt trauma patients admitted to the ICU at a Level I trauma center, prior study demonstrated that there was no difference between obese and lean patients in the type of traumatic brain injury [15]. Another study demonstrated similar injury patterns of fewer head but more chest and lower extremity injuries [16]. It has also been reported that obese trauma patients sustained more pelvic, rib, and lower extremity fractures but fewer liver injuries, mandibular fractures, and cerebral injuries than those non-obese trauma patients [17]. Based on our analysis of the AIS scores, obese patients presented no significant difference of injuries to body region from normal-weight patients. Comparison of the composition of AIS severity scaling from 1 to 5 in each region also did not show a significant difference between obese and normal-weight patients.

Longer hospitalizations of obese patients result in increased morbidity and are associated with impaired mobility, higher incidence of respiratory complications, more venous thromboembolic events, and higher nosocomial infection rates [18]. For example, obesity resulted in nearly twofold-increased odds for developing cardiac and pulmonary complications after a hip fracture as well as significantly increasing the odds of developing infectious complications (OR 3.8; 95 % CI 1.9–7.6, *p* < 0.001) [19]. It has been reported that the mean duration of orthopedic surgery in morbidly obese patients was 30 % longer than in non-obese patients [20]. Moreover, medically stable obese patients were found to be almost twice as likely to experience delayed fracture fixation due to preference of the surgeon [20]. In addition, obesity in critically ill patients is significantly related to a prolonged duration of mechanical ventilation and intensive care unit length of stay [21, 22]. In this study, although obese patients had a significant lower ISS than normal-weight patients who had experienced a fall, the obese patients had a significantly longer in-hospital LOS than did normal-weight patients. Even after taking account of possible differences in comorbidity and ISS, the obese patients have an average 1.54 day longer LOS than that of normal-weight patients.

Limitation of this study involves the use of a retrospective design with its inherent selection bias and the lack of available data on the circumstances of the mechanisms of injury. In addition, the patients dead on hospital arrival or accident scene are not included into the Trauma Registry Database, thus creating a selection bias. Moreover, some well-established risk factors, including prior falls, inappropriate use of medications, gait or balance problems, and functional limitations, were not documented and analyzed in this study. Finally, this study is only descriptive and, therefore, unable to assess the effects of any particular treatment intervention; it could only rely on the assumption that assessment and management are uniform between obese and normal-weight populations.

## Conclusion

The obese adult patients presented with similar injury to the body region following a fall in comparison with normal-weight patients. The obese patients had significantly lower ISS but significantly longer in-hospital LOS than did normal-weight patients. However, mortality, the percentage of patients admitted to the ICU, and LOS in the ICU exhibited no statistically significant differences between obese and normal-weight patients.

## Level of evidence

Epidemiological study, level III.
